# Resveratrol modulates phosphorylation of ERK and AKT in murine cementoblasts during in vitro orthodontic compression

**DOI:** 10.1186/s12903-025-05591-5

**Published:** 2025-02-13

**Authors:** Shams Negm, Michael Wolf, Rogerio B. Craveiro, Leon Schurgers, Joachim Jankowski, Rebekka K. Schneider, Marta Rizk, Franziska Coenen, Isabel Knaup, Sihem Brenji, Christian Niederau

**Affiliations:** 1 Department of Orthodontics, Medical Faculty, RWTH Aachen University, Aachen, Germany; 2 Department of Biochemistry, CARIM, University of Maastricht, Maastricht, the Netherlands; 3 Institute of Molecular Cardiovascular Research, Medical Faculty, RWTH Aachen University, Aachen, Germany; 4 Aachen-Maastricht Institute for CardioRenal Disease (AMICARE), University Hospital RWTH Aachen, Aachen, Germany; 5 Department of Pathology, Cardiovascular Research Institute Maastricht (CARIM), University of Maastricht, Maastricht, The Netherlands; 6 Department of Cell and Tumor Biology, Medical Faculty, RWTH Aachen University, Aachen, Germany

**Keywords:** OCCM-30, Orthodontic tooth movement, Compression, Inflammation, Orthodontically induced inflammatory root resorption, Sterile inflammation, Mechanical stimulation

## Abstract

**Objective:**

Resveratrol is a plant polyphenol known for its anti-inflammatory and pro-regenerative properties. These could be beneficial in controlling potential side effects of orthodontic treatment, such as apical root resorption. Orthodontic tooth movement occurs as part of a sterile inflammatory response. However, dysregulation of this process can result in pathologically increased osteoclast activity in the radicular vicinity, leading to unwanted root resorption. Previous studies have shown that root cementum cells can modulate recruitment of osteoclast precursors and cementum repair.

**Material and methods:**

We investigated the effect of resveratrol on mechanically stimulated murine cementoblasts (OCCM-30) with regards to cell viability, and mRNA expression and protein levels of pro-inflammatory cytokines. Furthermore, the modulation of central related kinases was investigated.

**Results:**

Resveratrol increased viability of OCCM-30 in a time- and dose-dependent manner and significantly reduced upregulation of pERK and pAKT, upstream regulators of key cellular metabolic pathways. Furthermore, we describe for the first time that cementoblasts respond to compression with accelerated activation of STAT3 and increased translocation of NF-κB p65 into the nucleus.

**Conclusion:**

This study shows a regulation of pAKT and pERK by resveratrol in OCCM-30 cells without a negative effect on cell viability. Therefore, resveratrol may have the potential to modulate the periodontal response to mechanical stimulation.

**Supplementary Information:**

The online version contains supplementary material available at 10.1186/s12903-025-05591-5.

## Introduction

The application of orthodontic force causes the tooth to move in any three-dimensional direction within its alveolar space [1]. By this, the periodontal ligament (PDL) is compressed in some areas and stretched in others. This mechanical stimulation of soft tissues induces an aseptic inflammation with complex metabolic cascades that allow periodontal remodeling and, consequently, tooth movement [2]. Under physiological conditions, remodeling involves a balanced interplay between tissue formation and degradation [3]. Because this process is not microbially induced and occurs in a regulated manner with simultaneous healing processes, orthodontic tooth movement is referred to as a sterile inflammatory response of the periodontium [4].

Complications in orthodontics are due to an imbalance or overreaction of this inflammatory response. The remodeling of the periodontium is disturbed, which can lead to gingival recession, bone loss and external root resorption [5]. Orthodontically induced inflammatory root resorption is a common side effect, occurring in approximately 80% of patients [6], with up to 15% of these cases showing advanced apical resorption of more than 4 mm [7]. It is hypothesized that induced inflammatory root resorption occurs as collateral damage when hyalinized areas within compression zone are removed [8]. The root undergoes pathological degradation by osteoclasts through the release of osteolytic enzymes [9]. Induced inflammatory root resorption predominantly affects those parts of the root surface exposed to high compressive stress, with the severity of resorption increasing in proportion to the applied force, leading to greater hyalinization and subsequent degradation [10].

It is proposed that under relatively strong orthodontic forces, cementocytes may contribute to the recruitment and activation of osteoclast precursors, by increasing RANKL (receptor activator of nuclear factor kappa-Β ligand) levels while decreasing OPG (osteoprotegerin) expression [11]. However, under moderate mechanical stimuli, root cementum cells are thought to exert protective effects; cementocytes reverse the RANKL:OPG ratio, thereby inhibiting induced inflammatory root resorption [12]. In addition, cementoblasts are responsible for some repair of resorption lacunae by depositing intrinsic fiber cementum when the compressive forces are reduced, or the application of force is interrupted [13].

Various clinical procedures have been developed to influence orthodontic treatment such as: corticotomy, laser therapy, vibration therapy and administration of biomodulators. These approaches aim to regulate the inflammatory response, in particular the activity of osteoclasts and osteoblasts, via the RANKL-OPG system. The goals are to adjust the rate of orthodontic tooth movement, improve biological anchoraging, ensure the stability of treatment outcomes and prevent complications [14, 15].

Resveratrol (3,4',5-trihydroxystilbene) has potential as a biomodulator in this context. It is a naturally occurring polyphenol that is widely distributed in plants, where it acts as a phytoalexin to protect against infections and stress [16]. It is most famously associated with red wine and the phenomenon known as the “French paradox”, which observed that despite a diet high in fat and alcohol, the French population had a lower incidence of cardiovascular disease. It was hypothesized to be due to resveratrol in wine [17, 18]. Although the hypothesis has not been confirmed [19, 20], interest in resveratrol has remained high, leading to extensive research into its effects. In vitro studies have shown that resveratrol has anti-inflammatory, antioxidant and immunomodulatory properties. It has demonstrated activity against tumor formation, osteoporosis [21], cardiovascular and neurodegenerative diseases [16].

The aim of this study is to investigate resveratrol as an anti-inflammatory and pro-regenerative biomodulator to potentially influence cementoblasts in periodontal compression zones and thus be used to mitigate induced inflammatory root resorption. In addition, cementoblast physiology under mechanical stress can be further elucidated.

## Material and methods

### Cell culture

All cell biology methods were performed under sterile microbiological conditions. To examine cement-forming cells in vitro, OCCM-30 cells, a cell line of immortalized murine cementoblasts, are well established in current research [22]. OCCM-30 cells were kindly provided by Prof. M. J. Somerman (NIH). OCCM-30 cells were cultured in DMEM low glucose cell culture medium (1 g/dL; Gibco, USA, #31885049), supplemented with 10% fetal calf serum (Gibco, USA, #10500–064) and 1% penicillin–streptomycin (10,000 U/mL; Gibco, USA, 15140–122). Conditions of 37 °C, 5% CO_2_ saturation and 95% humidity were met. The cells were trypsinized (trypsin–EDTA 0.05%; Gibco, USA, #25300–054), centrifuged (22 °C, 300 × *g*, 5 min) and quantified using the Neubauer counting chamber before plating: for the scratch assay 14 × 10^4^ cells and for the RT-qPCR, Western blot and immunofluorescence 18 × 10^4^ cells were plated in each 6-well, while 96-well plates and correspondingly 0.5 × 10^4^ cells were used for the MTS assay. After attaining a cell confluence of 90%, respectively 80% in the scratch assay, 10 µM resveratrol EP (Sigma-Aldrich, USA, #Y0001194), diluted in DMSO (Sigma-Aldrich, USA, #D4540), was administered. Additional concentrations were included in the MTS assay. Control groups were treated with DMSO alone.

### Static compressive stimulation

The model of Kanzaki et al. [23], in which cells are loaded with glass plates of defined area density, is a proven method to mimic the compression zone in vitro. It has already been successfully applied to OCCM-30 cells [24–27]. In this study, a static compressive force of 2 g/cm^2^ was exerted for a period of 16 h, once the cell monolayer reached 90% confluence and one hour after drug application. For the immunofluorescence, the pressure was administered for a shorter period of three hours.

### MTS assay

The CellTiter 96® AQ_ueous_ One Solution Cell Proliferation Assay (Promega, USA, #G3580) was used to determine the viability of OCCM-30 cells under the influence of resveratrol. Different drug concentrations, from 0 to 10 µM, were investigated. After 24, 48 and 72 h of exposure, 20 µL MTS was applied to each well for two hours, before detecting the absorbance at 490 nm using the NanoDrop One/One^c^ UV–Vis Spectrophotometer (VWR, USA, #732–2939). The amount of formazan dye, produced by the reduction of MTS through glycolysis of viable cells, was measured. Results were normalized to the mean value of the 0 µM control group. The data represent three experiments in biological quadruplicate.

### Wound healing assay via scratch

The OCCM-30 cell migration was assessed using a wound healing assay. One hour after substance application, a scratch was made in the cell monolayer using a 200 µL pipette tip. Images were captured at 0, 12 and 24 h after scratching using the Axio Observer 7 (Carl Zeiss, GER, #491917-0001-000) with the ZEN Pro microscope software (Carl Zeiss, GER). The gap width was measured at three different locations per image and averaged, before calculating the growth distance at 12 and 24 h. The data represent three experiments in biological duplicate.

### Isolation and purification of RNA

Through RT-qPCR the gene expression of *Il-6* and *Cox2* was assessed. Following drug and pressure application, the cells were reaped by washing each well with PBS (Gibco, USA, #14190–169) and lysing with 0.5 mL TRIzol Reagent (Thermo Fisher Scientific, USA, #15596–018). RNA isolation was performed as per the manufacturer’s instructions, before photometric examination of each sample, utilizing the NanoDrop. The yield and purity of RNA was verified by A260/A280 and A260/A230 ratio analysis. For RNA clean-up the Quick-RNA MicroPrep Kit (Zymo Research, USA, #R1051) was used, incorporating on-column DNA digestion through RNase-Free DNase Set (Qiagen, GER, #79254). To confirm successful purification each sample was re-measured by NanoDrop.

### Quantitative real-time PCR analysis (RT-qPCR)

The desired final concentration of 2.5 ng/μL cDNA was calculated from the amount of RNA found after purification. Synthesis was performed by reverse transcriptase of the isolated RNA, using the SuperScript III RT (Thermo Fisher Scientific, USA, #18080–044). To avoid experimental deviations, RNA isolation to cDNA transcription was performed in parallel for all samples of each experiment. The resulting cDNA was prepared using Luminaris Color HiGreen qPCR Master Mix (Thermo Fisher Scientific, USA, #K0394) and self-designed intron spanning primers, followed by RT-qPCR in technical duplicates. The qTOWER^3^ Real-Time-Thermocycler (Analytik Jena, GER, #844–00553-2) and the qPCRsoft 4.0 program (Analytik Jena, GER) were used. Primers were designed via Primer-BLAST (NCBI, USA) and controlled by a PCR check (Eurofins Oligo Analysis Tool, LUX) to ensure in silico qPCR specificity. Design criteria were: Length approximately 20 bp, annealing temperature 60 °C, maximum product length 200 bp, intron spanning, covering possible transcript variants. The RT-qPCR protocol consisted of initial steps of 50 °C/2 min and 95 °C/10 min, followed by 40 cycles of 95 °C/15 s, 60 °C/30 s and 72 °C/30 s. A subsequent interval of 95 °C/15 s provided transition to the melting curve analysis (60–95 °C). *Rpl22* was used as a reference gene as we have previously established it to be the most stable for mechanically stimulated OCCM-30 cells [26]. Detailed gene, primer and target/amplicon information is shown in Table 1. Mean Ct values were normalized to *Rpl22* and presented as fold change relative to the control. The data represent five experiments in biological triplicate and technical duplicate.

**Table 1:** RT-qPCR gene, primer and target/amplicon information for the reference gene *Rpl22* and investigated target genes

**Gene symbol**	**Gene name** (Mus musculus)	**Gene function**	**Accession number** (NCBI Gene Bank)	**Chromosoma location** (length)	**5’-forward primer-3’** (length/Tm/%GC)	**5’-reverse primer-3’** (length/Tm/%GC)	**Primer location**	**Amplicon length**	**Amplicon location** (bp of start/stop)	**Intron- flanking** (length)	**Variants targeted** (transcript/splice)
*Il-6*	Interleukin 6	Important role in bone metabolism; osteoclastogenesis	NM_031168.2	5 B1; 5 15.7 cM (1083 bp)	ACTTCACAAGTCGGAGGCTTA (21 bp/59.03 °C/47.62%)	TTTTCTGCAAGTGCATCATCGT (22 bp/59.45 °C/40.91%)	Exon 2/3	116 bp	220/335	Yes	Yes
*Ptgs (Cox2)*	Prostaglandin-endoperoxide synthase 2	Involved in prostaglandin synthesis	NM_011198.4	MT (non nuclear) (4460 bp)	TGAGTACCGCAAACGCTTCT (20 bp/59.97 °C/50%)	GCAGGGTACAGTTCCATGACA (21 bp/60 °C/52.38%)	Exon 9/10	126 bp	1543/1668	Yes	-
*Rpl22*	Ribosomal protein L22	Translation of mRNA in protein	NM_001277113.1	4; 4 E2 (2153 bp)	AAGTTCACCCTGGACTGCAC (20 bp/60.18 °C/55%)	AGGTTGCCAGCTTTCCCATT (20 bp/60.18 °C/50%)	Exon 2/3	110 bp	116/275	Yes	Yes

*Tm* melting temperature of primer/specific qPCR product (amplicon), *%GC* guanine/cytosine content, *bp* base pairs, *MT* mitochondrial

### Western blot

Protein expression of AKT, ERK 1/2, STAT3 and their phosphorylated counterparts were determined via Western blot. After resveratrol treatment and once the pressure has been relieved, the cells were harvested by RIPA-buffer (Thermo Fisher Scientific, USA, #89900), supplemented with protease inhibitors (cOmplete Mini; Roche Holding, CHE, #04693124001) and phosphatase inhibitors (PhosSTOP; Roche Holding, CHE, #04906837001). A total protein amount of 20 or approximately 25 µg (depending on whether a 15- or 10-well gel was used) was quantified by Bradford assay. The samples were separated by gel electrophoresis (TGX Stain-Free FastCast; Bio-Rad, USA, #1610185) and the proteins were transferred to a nitrocellulose membrane through western blotting. The Trans-Blot Turbo RTA Mini 0.2 µm Nitrocellulose Transfer Kit (Bio-Rad, USA, #1704270) was used. Membranes were blocked in a BSA solution: Tris-buffered saline and 0.05% Tween-20 (TBS-T; Carl Roth, GER, #2449.2 + #9127.1), added with 5% BSA (Carl Roth, GER, #8076.4). After incubation with the primary antibodies, the corresponding secondary antibodies were added, diluted in BSA solution.

The Primary antibodies were sourced from Cell Signaling, GBR: AKT (pan) (40D4) (1:2000; #2920), phospho-Akt (Ser473) (D9E) (1:2000; #4060), p44/42 MAPK (Erk1/2) (3A7) (1:1000; #9107), phospho-p44/42 MAPK (Erk1/2) (Thr202/Tyr204) (D13.14.4E) (1:2000; #4370), Stat3 (124H6) (1:1000; #9193), phospho-Stat3 (Tyr705) (D3A7) (1:2000; #9145) and GAPDH (14C10) (1:1000; #2118). Secondary antibodies were obtained from BioRad, USA: StarBright Blue 520 (1:3000; #12005867) and StarBright Blue 700 (1:3000; #12004162).

The immunoblot was fluorescently visualized, quantified, and normalized through ChemiDoc MP Imaging System (Bio-Rad, USA, #12003154), utilizing the Image Lab Software (Bio-Rad, USA) and Stain-Free technology. This approach is independent of endogenous proteins, as it measures the intensity of detected bands relative to the total protein in the lane. Hence, this method enables more precise protein quantification and enhances the accuracy of western blotting outcomes [28]. The adjusted band’s volume corrected by the normalization factor („Norm. Vol. (Int)“) is retrieved and the values are normalized to the respective control group. Six independent experiments were carried out.

### Immunofluorescence

The localization and nuclear translocation of the p65 subunit of NF-κB was visualized using immunofluorescence. Upon completed drug and pressure administration, the cells were fixated in 3.7% formaldehyde (in PBS; Carl Roth, GER, #7398.1), permeabilized in 0.1% Triton X (in 1% BSA solution in PBS; Carl Roth, GER, #3051.3) and then blocked with 1% BSA solution (in PBS; Carl Roth, GER, #8076.4). The target was detected by adding the primary antibody NF-κB p65 (D14E12) (1:400; Cell Signaling Technology, GBR, #8242) and the fluorescence-carrying secondary antibody Alexa Fluor 488 (1:200; Thermo Fisher Scientific, USA, #A-11034). Afterwards, the cytoskeletons were stained with rhodamine phalloidin staining (Thermo Fisher Scientific, USA, #R415). ProLong Gold Antifade Mountant with DAPI (Thermo Fisher Scientific, USA, #P36935) was applied to protect photobleaching of fluorescent dyes and to lable the nuclei. Microscopy was performed with the Axio Observer 7 and the ZEN Pro microscope software. The percentage of the area occupied by translocated NF-κB p65 relative to the total nucleus area was calculated. The data represent three experiments in technical quintuplicate. Since the activation of NF-kB and its translation into the nucleus is expected to occur within minutes to a few hours, we shortened the stimulation period for this experiment to 3 h.

### Statistical analysis

The graphs show the mean ± standard deviation (SD). Data were assessed for normality by the Shapiro–Wilk test and statistical significance was analyzed via one-way analysis of variance (ANOVA), followed by Tukey’s multiple comparison test, using Prism 9.0 (GraphPad Software, USA). A *p-*value < 0.05 was considered statistically significant.

## Results

### Resveratrol enhances cementoblast viability in a time- and dose-dependent manner

**Fig. 1:**
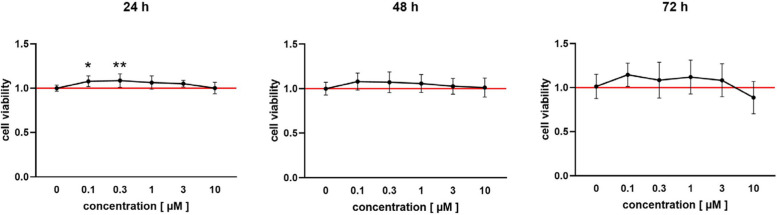
Effect of different concentrations of resveratrol, ranging from 0 to 10 µM, on OCCM-30 cell viability, by MTS assay. Exposure times were 24, 48 and 72 h. Data were normalized to the mean value of the 0 µM control group (red line). Values were expressed as mean ± SD of three passages in biological quadruplicate. Significance was calculated relative to the 0 µM control group. *p < 0.05 was considered statistically significant by one-way ANOVA. A viability-promoting trend can be seen, with significance at 0.1 and 0.3 µM after 24 h. Only after 72 h, 10 µM resveratrol has an insignificant cytotoxic effect

The effect of resveratrol on cementoblast viability and its cytotoxic potential were assessed using the MTS assay, considering different doses and treatment durations. The concentrations tested were based on the maximum plasma levels observed in a rat model, which were approximately 0.5 mg/L (equivalent to 2 µM) [29] and based on human plasma levels (0.024 – 25 µM) [30]. The lowest doses of 0.1 and 0.3 µM significantly improved OCCM-30 cell viability after 24 h of drug exposure. This beneficial effect was observed as a trend across all concentrations at different time points, except for the highest concentration. Treatment with 10 µM resveratrol did not improve cell viability and resulted in a non-significant decrease after 72 h. Overall, the viability-enhancing effects of resveratrol were most pronounced at shorter exposure times and lower concentrations, with no significant cytotoxic effects observed (Fig. 1).

### No effect of resveratrol on cell migration was observed

**Fig. 2:**
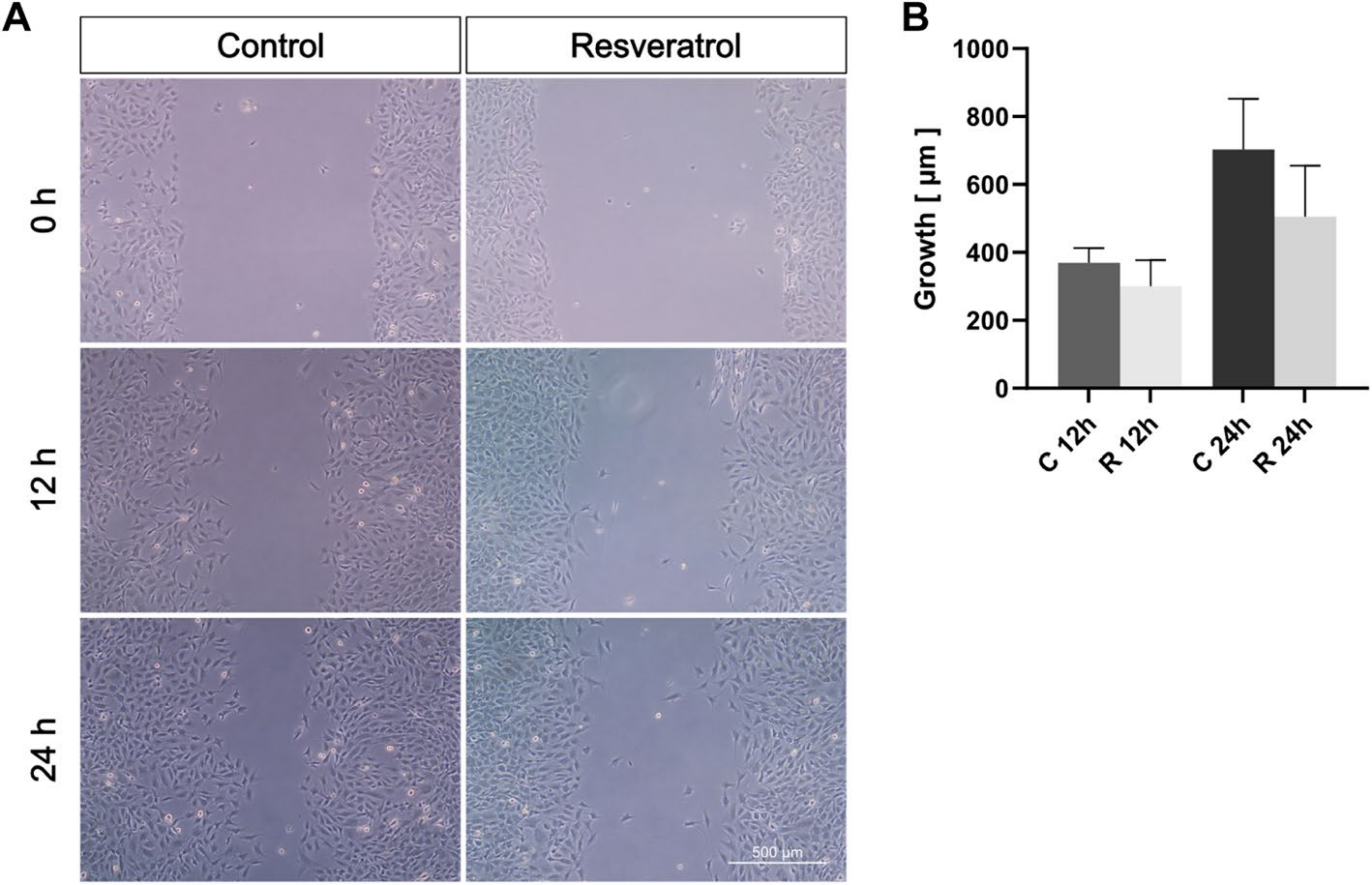
Effect of resveratrol on OCCM-30 cell migration, after 0, 12 and 24 h of wound healing, by scratch assay. Cells were treated with 10 µM. **A** Representative microscopic images of three passages are shown. **B** Quantification. Values were expressed as mean ± SD, of three passages in biological duplicate and technical triplicate. *p < 0.05 was considered statistically significant by one-way ANOVA. C = control. R = resveratrol. OCCM-30 cells migrate at a steady rate. No significant effects on cell migration were observed for resveratrol

We further analyzed the effect of 10 µM resveratrol on the proliferative dynamics of cementoblasts based on the scratch assay. Cell migration was assessed at 0, 12 and 24 h after scratching and the growth distances were calculated. Quantification showed that the control group had almost twice the migration distance at 24 h compared to 12 h. Thus, OCCM-30 cells migrated into the scratch at an approximately constant rate as part of simulated wound healing. Resveratrol had no significant effect on OCCM-30 cell migration; only a non-significant trend towards reduced regeneration rates can be seen, for both time periods (Fig. 2).

### Resveratrol tendentially inhibits the upregulation of Il-6 gene expression caused by mechanical stimulation

**Fig. 3:**
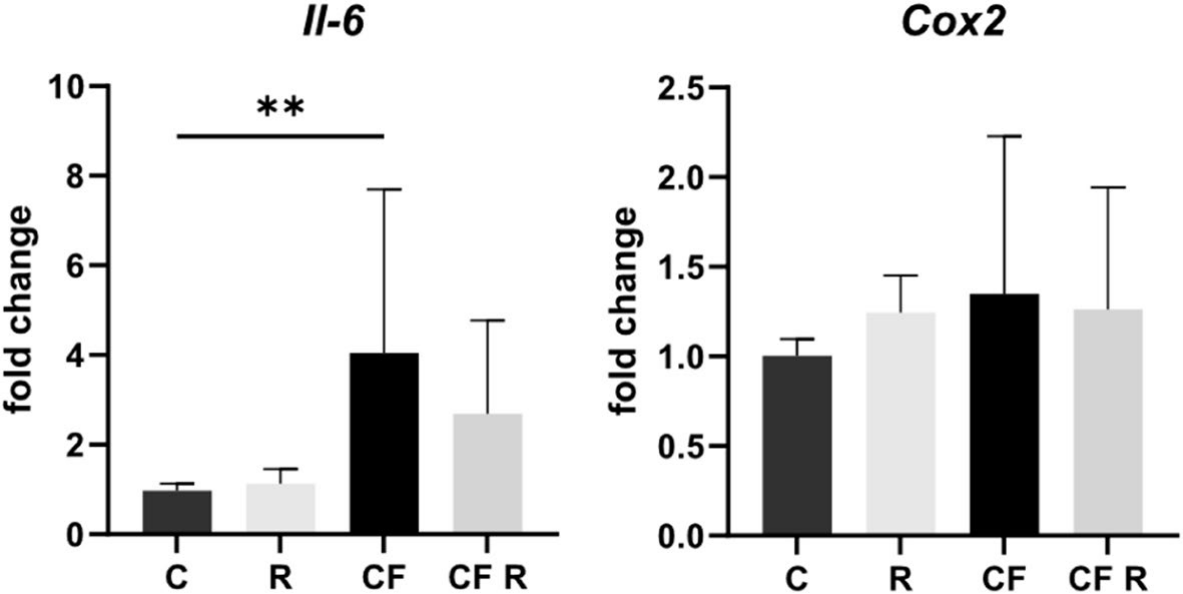
Effect of resveratrol on the gene expression of the target primers *Il-6* and *Cox2* in mechanically stimulated OCCM-30 cells, by RT-qPCR. Cells were treated with 10 µM drug followed by 16 h of pressure application. Data were normalized to the reference gene *Rpl22* and presented as fold of the control, which was set to 1. Values were expressed as mean ± SD, of five passages in biological triplicate and technical duplicate. *p < 0.05 was considered statistically significant by one-way ANOVA. C = control. R = resveratrol. CF = compressive force. *Il-6* expression increases significantly with pressure, whereas *Cox2* is not affected. Resveratrol reduced the upregulation of *Il-6* but did not significantly alter either marker

RT-qPCR was used to investigate the effects of 16 h compression and 10 µM resveratrol on the gene expression of *Il-6* and *Cox2* in OCCM-30 cells. Cementoblasts responded to pressure with a significant upregulation of *Il-6*. In addition, resveratrol reversed the increased expression of *Il-6*, but only in tendency and not down to control levels. However, it had no effect on basal gene expression. For *Cox2*, the increase with compressive force is not significant and has a high standard deviation. There are no effects of resveratrol on its basal or pressure-influenced expression (Fig. 3).

### Resveratrol inhibits ERK and AKT phosphorylation induced by compressive stimulation of OCCM-30 cells

**Fig. 4:**
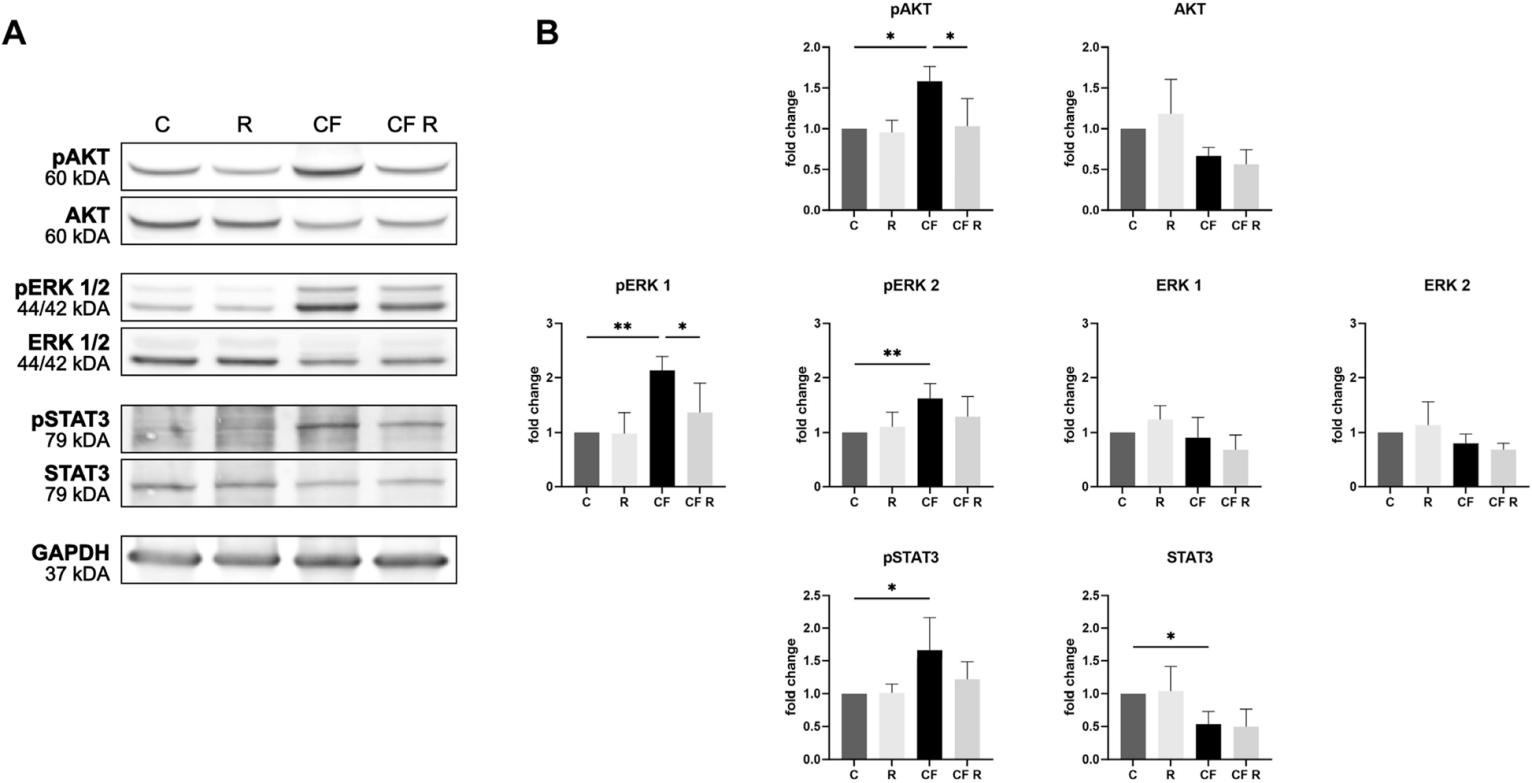
Effect of resveratrol on the expression and phosphorylation of the target proteins ERK 1/2, AKT and STAT3 in mechanically stimulated OCCM-30 cells, by Western blot. Cells were treated with 10 µM drug followed by 16 h of pressure application. **A** Representative blots from six passages are shown. GAPDH was used as reference. (See Supplementary Fig. S1 online for uncropped blots) **B** Quantification of blots and normalization using Stain-Free technology. Data were normalized to the control. Values were expressed as mean ± SD, of six passages. *p < 0.05 was considered statistically significant by one-way ANOVA. Phosphorylated variants are indicated with "p-". C = control. R = resveratrol. CF = compressive force. Compressive stimulation significantly increased pAKT, pERK 1/2, and pSTAT3. Resveratrol treatment significantly reduced pAKT and pERK 1, with a tendency to reduce pERK 2 and pSTAT3, but had no effect on basal protein expression

We investigated the expression and phosphorylation of the signal transduction proteins ERK 1/2 and AKT using Western blot analysis. Phosphorylation of ERK and AKT is not altered in the absence of compressive force, but 16 h of compressive stimulation at 90% cell confluence resulted in a significant increase in phosphorylated ERK 1/2 (pERK 1/2) and AKT (pAKT). In addition, treatment with 10 µM resveratrol under compressive stimulation significantly downregulated pERK 1 and pAKT, with pAKT levels returning to baseline conditions. pERK 2 also showed a decrease, but this was not statistically significant. The inactive forms of these proteins tended to decrease with increasing compression, consistent with the observed increase in phosphorylation. GAPDH levels were consistent across all samples, indicating reliable normalization (Fig. 4).

### Mechanical stimulation enhances STAT3 activation in cementoblasts

We also measured the protein levels of STAT3 by Western blot. Compressive force induced a significant activation of STAT3 in OCCM-30 cells, with an increase in its phosphorylated form and a decrease in its non-phosphorylated forms. Furthermore, resveratrol exhibited a tendency to downregulate STAT3 activation; however, it did not affect the basal expression levels of STAT3 or phosphorylated STAT3 (pSTAT3) (Fig. 4).

### OCCM-30 cells respond to compressive stimulation with nuclear translocation of NF-κB p65

**Fig. 5:**
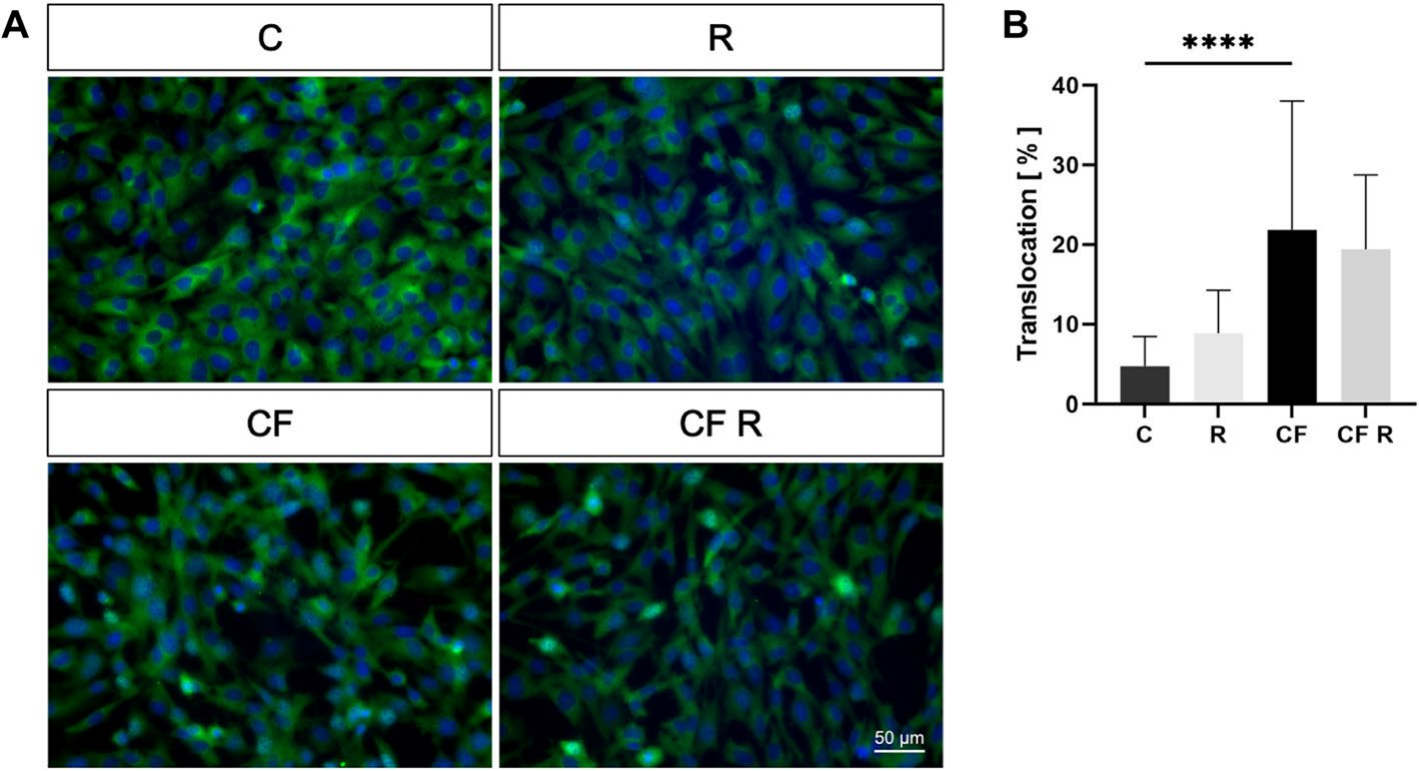
Effect of resveratrol on the translocation of NF-κB p65 into the nucleus of mechanically stimulated OCCM-30 cells, by immunofluorescence. Cells were treated with 10 µM drug followed by compressive stimulation for 3 h. **A** Representative microscopic images of three passages are shown. Cell nuclei (blue) and NF-κB (green) are shown. **B** Quantification of the NF-κB p65 portion of the nuclei is shown as percentage. Values were expressed as mean ± SD, for three passages in technical quintuplicate. *p < 0.05 was considered statistically significant by one-way ANOVA. C = control. R = resveratrol. CF = compressive force. A significant translocation of NF-κB p65 was observed after compressive stimulation, indicating an inflammatory response of OCCM-30 cells. Resveratrol has no significant effect on NF-κB p65 localization under either basal or compressive conditions

The localization of NF-κB p65 in weight-loaded cementoblasts was investigated. Its translocation from the cytoplasm to the nucleus, indicating its role as a transcription factor, was observed by immunofluorescence, under the influence of three hours of compressive stimulation and 10 µM resveratrol. Representative microscopic images show a high translocation of NF-κB p65 after mechanical stimulation. This observation was confirmed with significance by quantification. Resveratrol did not significantly affect the localization of NF-κB p65, either under basal conditions or following compressive force application (Fig. 5).

## Discussion

Resveratrol is a naturally occurring polyphenol, known for its anti-inflammatory [31], antioxidant [32], immunomodulatory [16] and osteoprotective [21] properties. In our in vitro study, we examined its effects on immortalized cementoblasts, which were mechanically loaded to simulate compressed PDL areas during orthodontic tooth movement. The central question was whether resveratrol could modulate the inflammatory processes induced by mechanical stimulation and thereby potentially minimize the side effects of orthodontic treatment, such as induced inflammatory root resorption [33].

Resveratrol concentrations used in this study were based on those found naturally in red wine (1.5—3 mg/L, equivalent to 6.57—13.14 µM) [34] and in human blood plasma (0.024—25 µM) [30]. Other studies with periodontal cells and resveratrol have so far used significantly higher concentrations of up to 100 µM [35, 36]. Following the MTS assay, we decided to use the highest concentration tested, 10 µM, for further experiments as this concentration also showed no cytotoxic effects over the planned duration of action. After oral intake, it is rapidly absorbed and metabolized in the small intestine [37], resulting in an oral bioavailability of about 12% trans-resveratrol [38]. As drugs would be administered locally in the periodontium during orthodontic therapy, plasma levels and systemic bioavailability are negligible in the present case.

Resveratrol is well known for its low toxicity profile. It has low acute and chronic toxicity in rats up to a dose of 1,000 mg/kg body weight, while short-term human studies show only minor and inconsistent side effects at high doses, such as gastrointestinal upset at 2.5 or 5 g of resveratrol per day [39]. There are no data on long-term use in humans. For our experiments, we chose a concentration of 10 µM as this has shown to be the most effective on protein and mRNA expression, without showing cytotoxic activity, according to the MTS assay.

The method of compressive stimulation was based on previous studies on human periodontal ligament cells (hPDL) [40, 41] and cementoblasts [24–27].

We found that cementoblasts respond to mechanical stimulation with a four-fold increase in *Il-6* and a trend towards upregulation of *Cox2* mRNA. This is in line with our previous publications [25, 26]. Furthermore, we are the first to observe that cementoblasts respond to compression with STAT3 activation and a 5.5-fold increase in the nuclear translocation of NF-κB p65. The involvement of STAT3 in tooth movement has already been demonstrated histologically in an in vivo model by Jin et al. [42]. The increased gene expression of *Il-6* and *Cox2*, together with the increased activation of the transcription factors STAT3 and NF-κB, indicate an inflammatory response of cementoblasts in the compressive zone [25, 43, 44]. This suggests that cementoblasts are involved in the remodeling process of the periodontium that ultimately enables tooth movement [45]. Furthermore, Diercke et al. [46] deduced COX-2-dependent *Rankl* mRNA expression in mechanically stimulated cementoblasts – which is not compensated by increased *Opg* levels. IL-6 is also known to play a crucial role in RANKL induction [47].

In previous work, we have shown that phosphorylation of ERK and protein kinase B (AKT) is increased under compressive forces and is dependent on cell density and the duration of the compressive stimulus [27]. In this study, we were able to show that at 90% cell confluence, pAKT and pERK 1/2 were again upregulated at longer compression times of 16 h. These phosphorylations are known modulators of cell metabolism involved in cell proliferation, growth and survival [48, 49]. Furthermore, Ye et al. [50] reported that the chemerin/ChemR23-induced TNF-α and IL-6 expression of OCCM-30 cells depends on the activation of the ERK 1/2 and PI3K-AKT signaling pathways. Similarly, Pan et al. [51] found that the blockade of the NF-κB pathway reduced IL-6 expression. This suggests that an inflammatory response of cementoblasts to mechanical compression leads to an increased expression of IL-6, via the activated signaling pathways of NF-κB, ERK 1/2 and PI3K-AKT.

Our study also aimed to investigate the potential of resveratrol in preventing induced inflammatory root resorption, an adverse side effect caused by dysregulated inflammation in the event of pathological compressive stress of the PDL [5]. Since the cementoblasts can repair limited resorption of the tooth root following mechanical stimulation [13], the viability-enhancing effects of resveratrol on these cells are of interest if such resorption is to be reduced. We found a time- and dose-dependent viability-promoting effect of resveratrol on cementoblasts; especially at shorter exposure times and lower concentrations. In contrast, resveratrol did not show a positive effect on cell proliferation. This discrepancy requires further investigation of the mechanisms by which it affects cell viability while apparently not promoting growth kinetics.

Resveratrol affects several molecular signaling pathways, including SIRT1, NF-κB, antioxidant enzymes, RANKL, COX and IL-6 [16]. One of its major targets appears to be SIRT1 [52], a deacetylase that modulates the activity of several regulators such as NF-κB [53], AKT [54], ERK [55] and STAT3 [56]. Pannu and Bhatnagar [16] described that the modulation of SIRT1 and AKT is key in the regulation of autophagy by resveratrol. As part of its anticarcinogenic effects, resveratrol inhibits nuclear translocation of NF-κB, the expression of iNOS and phosphorylation of AKT in colon tumors [57]. It also reduces the activity of ERK and STAT3 in cervical cancer cells [58]. According to our results, resveratrol reversed the pressure-induced activation of ERK 1 and AKT in cementoblasts and restored pAKT to control levels. No other effects were observed. Decreases in pERK 2 and pSTAT3 were not significant*.* The nuclear translocation of NF-κB p65 was also not significantly affected.

According to Szewczuk et al. [59] resveratrol exerts its anti-inflammatory effects mainly by inhibiting NF-κB, suppressing COX-1 activity and reducing prostaglandin synthesis. It also inhibits the production of MCP-1, TNF-α, IL-1β and IL-6 [60]. In our study, resveratrol did not show similar effects on cementoblasts. *Cox2* expression was not affected either at baseline or under pressure. Similarly, the increased expression of *Il-6* was only reduced by trend and not to basal level. Resveratrol also had no significant effect on the translocation of NF-κB p65. However, it must be taken into account that these studies were carried out with completely different cells and not with a mechanical stimulation model. This could explain the deviating results of our study.

In contrast, previous studies found that resveratrol has an anti-inflammatory effect on human periodontal fibroblasts, after combined mechanical compression performed as in our study and hypoxia [61, 62]. They demonstrated significantly reduced expression of the pro-inflammatory cytokines IL-6, IL-8, TNF-α and VEGF. This data represents a promising option for modulating inflammatory responses in the periodontium which could help combat orthodontic side effects. The effect of resveratrol on hPDL is crucial because periodontal fibroblasts play a major role in tooth movement and are also important for cementogenesis in terms of the production of Sharpey fibers [63]. hPDL are significantly involved in periodontal remodeling, inducing angiogenesis, inflammation, bone remodeling and matrix reorganization [64]. In this context, resveratrol does not seem to have a negative effect on the important interaction between PDLSC and cementoblasts, as no negative effect on cell viability could be detected. Therefore, resveratrol can be used to modulate the inflammatory response of PDLSC without affecting cementoblasts.

## Conclusion

We found that resveratrol inhibited pressure-induced phosphorylation of the ERK and AKT pathways in cementoblasts, while promoting cell viability in a dose- and time-dependent manner. This may provide a basis for using the known effects of resveratrol in PDLSC cells and the periodontal ligament to modulate the periodontal response to mechanical stimulation without having negative effects on cementoblasts.

## Supplementary Information

Supplementary Material 1.

## Data Availability

The data that support the findings of this study are available from the corresponding author upon reasonable request.

## Abbreviations

OCCM 30: Murine cementoblast cell line
IL-6: Interleukin 6
COX2: Cytochrome c oxidase subunit 2
ERK: Mitogen-activated protein kinase
pERK: Mitogen-activated protein kinase, phosphorylated
AKT: AKT serine/threonine kinase
pAKT: AKT serine/threonine kinase, phosphorylated
STAT3: Signal transducer and activator of transcription 3
pSTAT3: Signal transducer and activator of transcription 3, phosphorylated
RT-qPCR: Quantitative real-time PCR analysis
NF-κB p65: Nuclear factor kappa B
GAPDH: Glyceraldehyde-3-phosphate dehydrogenase
