# Supplementary material for: Resveratrol modulates phosphorylation of ERK and AKT in murine cementoblasts during in vitro orthodontic compression
Source: BMC Oral Health. 2025 Feb 13;25:226. doi: 10.1186/s12903-025-05591-5 (PMC11823246; doi:10.1186/s12903-025-05591-5)
Supplement: Supplementary file 1 — Supplementary Material 1. [file 12903_2025_5591_MOESM1_ESM.pdf]

Supplementary Data for the Paper:
"Resveratrol: influence on the inflammatory response and regenerative capacity of murine cementoblasts during *in vitro* orthodontic compression"

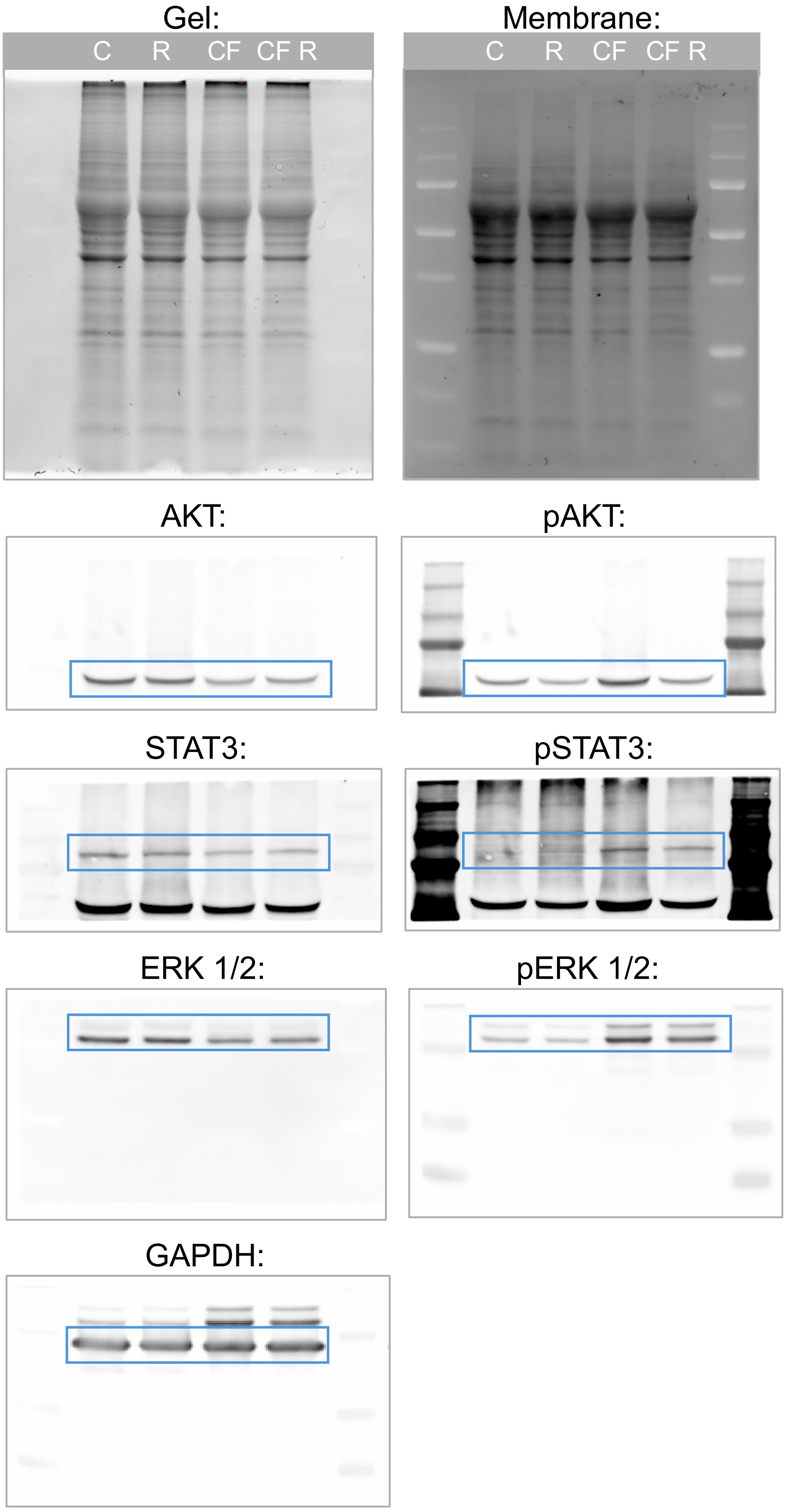

Fig. S1: Uncropped gel and blots of the Western blot.
C = control. R = resveratrol. CF = compressive force.
The published excerpts are marked in blue.
